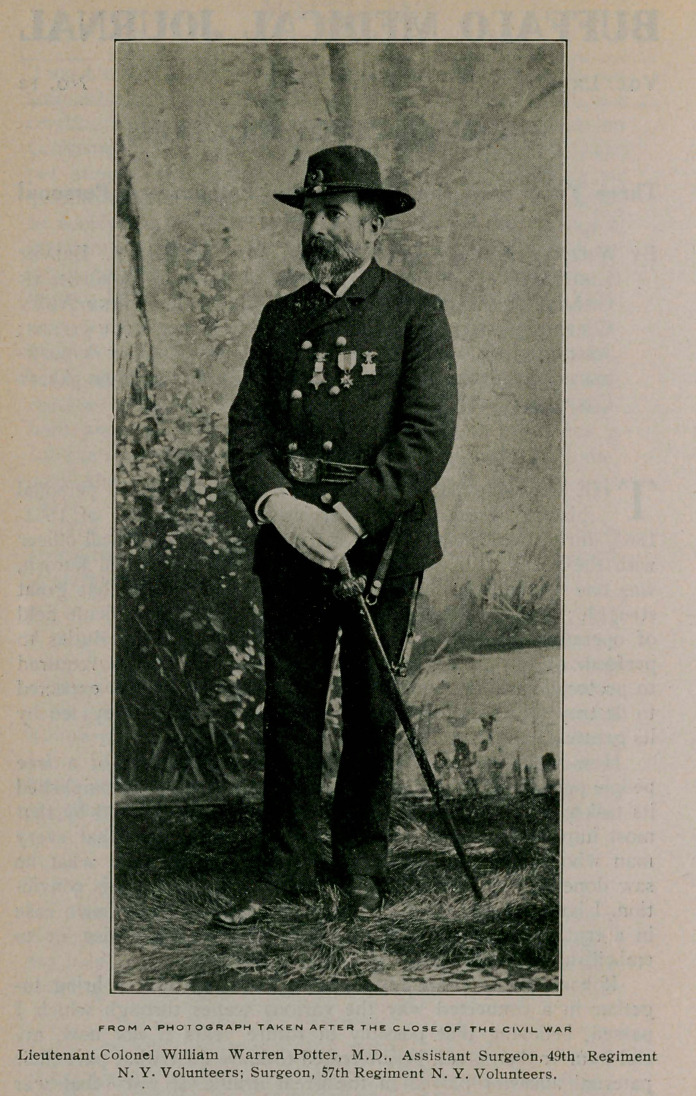# Three Years with the Army of the Potomac—A Personal Military History

**Published:** 1911-07

**Authors:** William Warren Potter

**Affiliations:** Buffalo, N. Y., Brevet Lieutenant Colonel, U. S. Volunteers; Surgeon in Charge of First Division Field Hospital Second Army Corps; Surgeon 57th Regiment New York Volunteers; Assistant Surgeon 49th Regiment New York Volunteers; Recorder Second Division Hospital Sixth Army Corps, etc., etc.


### BUFFALO MEDICAL JOURNAL

VOL. LXVI. JULY, 1911. NO. 12

### ORIGINAL COMMUNICATIONS

### Three Years with the Army of the Potomac—A Personal Military History

BY WILLIAM WARREN POTTER, M.D., BUFFALO, N. Y., BREVET LIEUTENANT COLONEL, U. S. VOLUNTEERS; SURGEON IN CHARGE OF FIRST DIVISION FIELD HOSPITAL SECOND ARMY CORPS; SURGEON 57TH REGIMENT NEW YORK VOLUNTEERS; ASSISTANT SURGEON 49TH REGIMENT NEW YORK VOLUNTEERS; RECORDER SECOND DIVISION HOSPITAL SIXTH ARMY CORPS, ETC., ETC.

### PREFACE

THE following pages are, as their title indicates, a personal history of my military service in the civil war of 1861-1865, during which time I served three years as a medical officer with the Army of the Potomac. That army, as is well known, was one of the two chief armies of the Republic in that great struggle; it had, as equally well known, the most difficult field of operation, and, as a consequence, the most trying duties to perform of any of the Union forces. It was always required to protect Washington, while, at the same time, it was expected to destroy the most formidable army of the Confederacy, led by its greatest captain.

How well that mighty creation of the patriotism of a free people performed its duties, or how satisfactorily it accomplished its tasks, is not for me to say—these will be determined by that most impartial tribunal, History—but it is expedient that every man who can do so, should record what he did, and what he saw done, during his military service. Acting upon this conviction, I have attempted to carry out this purpose in my own case in a truthful manner, without any attempt at self-praise, or to embellish the pages with anecdote or eulogy.

It has, on the other hand, simply been my aim to bring together in a connected way the various scenes through which I passed, believing that possibly in future years if not now, my children, or theirs, will be interested to know just what part their paternal ancestor played in the great drama of war—that war waged for a mightier principle than other in the history of the world.

FROM A PHOTOGRAPH TAKEN AFTER THE CLOSE OF THE CIVIL WAR

Lieutenant Colonel William Warren Potter, M.D., Assistant Surgeon, 49th Regiment N. Y. Volunteers; Surgeon, 57th Regiment N. Y. Volunteers.

The account is made up from letters which I wrote to my wife from the field that were always in the nature of bulletins, from memoranda made at the time, and from an undimmed recollection of every event narrated. The dates are believed to be absolutely accurate, and the impressions (where any are given), are such as obtained at the time. It is probable that, in the light of subsequent history, some of them may appear erroneous, or ill-conceived; but I prefer to let them stand as recorded at the time, as simply a reflex of the then accepted opinions of passing events.

In such a purely personal narrative the constant employment of the first person is unavoidable; yet, as it is not intended for publication, but merely for the eyes of relatives, or intimate friends, this will, I trust, be considered pardonable.

Finally, the consciousness of having performed my duty faithfully and to the utmost of my ability, even though humbly, during the period embraced in this record, has always been to me a comforting reflection, compensating in a full measure for "the dangers I had passed;" and if any one should, perchance, enjoy a moiety of the pleasure in the perusal of these pages that I did in preparing them, I shall be more than gratified.

W. W. P.

March 23, 1888.

---

### Three Years With the Army of the Potomac.

On the 15th day of April, 1861, three days after the fall of Fort Sumter, the President called for 75,000 men and, in apportioning the number from each state, New York's quota under this call and a second one made May 3, was fixed at 38 regiments of infantry. To raise, arm, equip, officer and get this force ready for the field, became the duty of the Governor (Morgan) and his military staff. His Surgeon General, Dr. S. Oakley Vanderpoel, at once issued a notice to medical men throughout the state who might desire to serve as surgeons and assistant surgeons, to assemble in Albany on the 25th and 26th days of April, 1861, for examination as to their fitness for these positions in the volunteer forces then raising.

At this time I was residing in Cowlesville, Wyoming Co., where I had been practising medicine in partnership with my uncle, Dr. Milton E. Potter, for about two years. I went to Albany on the 25th of April, presented myself for examination before the board of examiners in the assembly chamber next morning, completed my duties at two o'clock P. M., and left for home that night. In about two weeks I received notice that I had passed the examination, and would be appointed an assistant surgeon upon my nomination as such by the colonel of a regiment. It appears that under an old militia law rule this function was vested in the colonels of regiments, and this practice prevailed during the organisation of the first 38, or two years' regiments that the state of New York furnished. In all regiments subsequently raised the appointment of the regimental staff was made by the Governor direct, without the intervention of any preliminary nomination.

During the few weeks next following my successful examination, I made energetic though futile endeavors to obtain an appointment; but the numbers seeking places were so great, and the openings so few, that I soon began to despair of success. Most of these early appointments were obtained by men of influence with the several colonels, men much older and more experienced in affairs than I; for, be it known, I was at this time but 22 years of age.

Finally came the Bull Run disaster of July 21, 1861, and immediately thereafter followed a requisition upon our governor for 25,000 men for three years, under the authority of congress vesting power in the President to accept the services of 500,000 men. Buffalo had already, under the first call, sent out the 21st regiment for two years, and soon after the second call authority was vested in a coterie of patriotic citizens, under the name of the Union Defense Committee, to raise another regiment. This committee numbered among its members the Mayor, Hon. F. A. Alberger, James Adams, Dr. Edward Storck, Alderman A. A. Howard, Isaac Holloway, and others whose names I do not now recall.

Recruiting for the new regiment began on the 30th of July under the direction of this energetic committee. Fort Porter was selected as the rendezvous, and Major D. D. Bidwell appointed to command the post and superintend the recruiting. Major Bidwell was at the time an experienced officer in the State Militia, and, as Commandant of company "D" had attained a reputation as an efficient drillmaster, as well as a disciplinarian of an unusal order in civil life; and it was expected that he would be chosen to command the regiment whenever it should take the field. He selected for his assistant, George W. Johnson to act as major, with Henry D. Tillinghast as quartermaster, William Bullymore as adjutant, and the usual non-commissioned staff.

### Fort Porter.

Early in August I sought Major Bidwell's acquaintance, through his brother, Charles, whom I knew very well, and was authorised to obtain recruits for the command. I visited a number of the villages of Wyoming County, where the meetings were held, and succeeded in the course of a month in enlisting 10 or 12 men altogether, who were assigned to Company D which William F. Wheeler, now a lieutenant in the 21st regiment, was expected to command. On the 9th of September Dr. James A. Hall, of Brocton, Chautauqua Co., N. Y., was appointed surgeon of the regiment; for by this time the command had began to assume the dignity of that title, being designated for the sake of convenience, the Second Buffalo Regiment. I had already, during the period mentioned, visited the post several times with recruits, and on the morning of September 16, I left home with 2 or 3 more men. Arriving in the camp, to my astonishment, I found the regiment under orders to proceed to New York at once; so the remainder of the day was spent in making such hasty preparations for the journey as time permitted, 6 P. M. having been fixed upon as the hour for our departure.

Up to this time none of the field or staff officers had been appointed, with the single exception of Dr. Hall, as mentioned; but all determined to go to New York, myself among the number, in the expectation that we would there receive our commissions in the several places we desired. During the day I visited several of the Buffalo physicians and obtained from them recommendations to the Governor, hoping they might have influence in securing for me the appointment as Assistant Surgeon. I had already forwarded to the Governor, letters from Judge J. B. Skinner, Judge J. G. Hoyt, Hon. Augustus Frank, Albert Sawin and others, strongly recommending me for the place; but I sought to still further fortify my case in the manner described, believing that "thrice is he armed" who has plenty of indorsements from prominent public men.

We took up our march from Fort Porter about 4 P. M. on the day in question, which was Monday of the week, passing down Niagara to Main, thence to Exchange Street and the Erie Depot, where a special train was in waiting to convey us joyously on our mission. All the way from the Fort to the station the streets were crowded with people cheering us as we marched along, the houses and other buildings along the whole route being filled with interested spectators of the scene, flags and other emblems were displayed, shouts of encouragement went up, and a hearty God-speed came from thousands of throats. It was truly an ovation and a scene never to be forgotten. And so we were off for the seat of war, for this indeed was the first stage of our journey with that objective, amidst the booming of cannon, strains of patriotic music, and the huzzas and tears of our friends, the good citizens of Buffalo.

Our train was a heavy one and moved slowly, but we reached New York on the morning of the 18th of September without accident or mishap. We were accompanied by some of the members of the Union Defense Committee, others going direct to Albany to confer with the Governor, and finally meeting us in New York. Those who went to Albany reached New York on the evening of the 17th, and met us at the ferry in Jersey City next morning. In New York the company officers and men were quartered in Park Barracks, and the prospective field and staff at the Astor House, which was directly opposite the barracks. On our arrival at Jersey City, Dr. Storck of the Union Defense Committee, who had gone via Albany, met me with the information that my letter of appointment as assistant surgeon had been sent to Cowlesville by the surgeon general, that on reaching New York I was to report to that officer at 51 Walker Street, the New York headquarters for the organisation of troops, and there write my letter of acceptance.

After getting breakfast I so reported, and met Dr. Vanderpoel for the second time (the first having been the previous April when I was in Albany for examination) who received me with the utmost kindness, and informed me that as soon as he discovered the regiment had been ordered to New York he, finding my credentials of first class order, immediately wrote my letter of appointment and sent it Cowlesville, as before stated. He further said he was glad I had come on with the troops instead of waiting for the letter, and, as they would be sent on to the seat of war so soon, I could not be permitted to return home for a few days as I desired. I thus found myself suddenly taken from civil life and placed in the medical staff of the volunteer army, to which work I was to devote the next three years of my life, with what success the following pages will disclose.

My affairs at home were necessarily left in a somewhat chaotic state, due to the suddenness of my departure; but, thanks to my good wife, they were straightened out in due time, reflecting great credit upon her business sagacity, as well as patriotic devotion to husband, country and duty. For the next few days the state officials and the Union Defense Committee were busily engaged in organising and equipping the regiment, and by Saturday night, September 21, we were on our way to Washington.

### The Forty-Ninth.

I purchased a uniform in New York with money kindly loaned me by my good friend Alderman A. A. Howard of the Union Defense Committee, and I repaid him from the first money I received from the United States, sometime in November following. The regiment was designated the 49th New York Volunteers, and was officered as follows, viz: D. D. Bidwell, Colonel; W. C. Alberger, Lieutenant Colonel; George W. Johnson, Major; William Bullymore, Adjutant; Henry D. Tillinghast, Quartermaster; James A. Hall, Surgeon and William Warren Potter, Assistant Surgeon.

We reached Philadelphia about two o'clock A. M., Sunday, via the Camden & Amboy route, and were fed, even at that late hour, at the celebrated "Cooper Shop"—a refreshment establishment voluntarily maintained by the patriotic men and women of the city of brotherly love—the tables being served, even at that unseemly hour, by fair maidens and good young men, whose praises have been sounded by thousands of weary soldier-travelers on their way to the front. In the early morning air we marched across the city to the Baltimore Depot, and near eight o'clock A. M., were off for the latter city, which we reached about three P. M. Here we again marched across the city to the Calvert Station, and while doing so my mind was filled with thoughts of the "Plug Uglies" who had so murderously assaulted the Massachusetts 6th, on the 19th of April previously. However, we passed through the city very quietly, and were soon on our way to Washington, reaching the latter city about seven o'clock P. M.

The men were quartered for the night in the "Soldiers' Rest" adjoining the Baltimore & Ohio R. R. Depot, but the officers were obliged to remain in the waiting room of the station all night, without blankets or other articles of comfort. Here was begun the soldiers' experience of "roughing it", for it was truly a most uncomfortable night. We were here joined by Lieutenant Colonel Alberger, late captain in the 21st Regiment, who, with Dr. Wilcox, the surgeon of the 21st, was waiting for us at the depot on our arrival. The sight of one familiar face—some one to meet us at the train—was truly a refreshing circumstance, after a week of such experience as we had passed through. Next morning, Monday, September 23, we received orders to proceed out to Meridian Hill north of Washington, but yet within its outskirts, and there take up our camp. I strolled around the streets of Washington for an hour in company with Lieutenant Colonel Alberger, who pointed out the various objects of interest; after which I accepted his invitation to ride out to camp in a carriage with him—a luxurious mode of travel not usually enjoyed by a soldier. We arrived in camp about eleven o'clock A. M., and at noon had our first meal, such as it was, cooked in the field. We had not yet reached that degree of perfection in our culinary arrangements, which would enable us to rival

Delmonico's. In the afternoon I went with some of the other officers across Seventh Street Road, on which our camp fronted, and engaged board at Mr. Benjamin Summy's, an old resident of Lancaster; so we fared well while we remained at Meridian Hill. This family knew my wife's relations well, and inquired interestedly after them. Our comfort here was, however, destined to be of short duration, lasting only from Monday to Friday when "Camp Leslie"—for such it was called—was broken up. At noon, September 27, an order came for the regiment to report forthwith to Camp Lyon, located on the Maryland side of the Potomac about five miles above Georgetown. I was detailed to accompany the sick which were carried in two ambulances, and I set out about four o'clock P. M., by another route to overtake the regiment. The driver lost his way, so it was after dark, and in the midst of a pouring rain, when we reached Camp Lyon. Here I learned the regiment had been ordered across the Potomac whither it had gone; but, owing to the lateness of the hour, it was considered unwise to follow with the ambulances, so I sought quarters for the night with the 6th Wisconsin Regiment, whose surgeon, good Samaritan that he was, kindly "took me in." After breakfast next morning we started once more in search of the 49th, which we soon found near Fort Ethan Allen, about half a mile above Chain Bridge in Virginia. The roads were all filled with troops pouring into Virginia; but soon everything was straightened out, and the 49th was joined at first to General W. F. Smith's brigade, afterwards to General Isaac I. Stevens' brigade which formed a part of Smith's command, who had become the Commandant of a division. At last, after much fatigue of body and tribulation of spirit, I found myself on the "sacred soil" of Virginia, and our regiment in a division that was destined to make itself famous on the field of honor, in defense of the security of the Republic.

The regiment was put into camp south of the Fort under its guns, and furnished daily a large detail of men to work upon its unfinished walls. Here we had daily drills and frequent night alarms; during the latter we would go inside the Fort, sometimes for a few hours only and again remaining all night, expecting the enemy to attack; but he never came. One night, however, very soon after we came here, there was some firing in our front that occasioned great anxiety; but next morning we learned that the 72d, Pa., had, by mistake, fired into its own ranks, killing and wounding several men.

On the morning of October 2d at three o'clock, I started out with a picket detail from our regiment of two hundred and eighty men under the command of Captain Wheeler, who had already seen service of this kind in the 21st. As this was the first experience of the 49th of this sort, it was naturally an event of much moment to us; and I went along because it was customary to send an assistant surgeon with the picket detail to look after any who might fall sick or be wounded. The picket headquarters were near Langley Church on the Leesburg Turnpike, and we remained on this duty twenty-four hours. I visited the whole line on foot, as our horses had not yet arrived; and as it was about two miles long, I was very tired when night came. During the day W. H. Russell, the celebrated correspondent of the London Times who had written a sensational account of the Battle of Bull Run for his paper, visited Langley and I saw him for the first and only time. Governor Patterson of Chautauqua County had visited us the day before (Tuesday) at our camp, and complimented the regiment upon its soldierly appearance. We returned to camp Thursday morning, October 3, much fatigued after our tour of picket duty, for our march was some five miles after we were relieved. The experience was, however, wholesome and served to give us confidence in ourselves.

October 12, the regiment, excepting two companies, moved forward from Fort Ethan Allen about five miles to "Camp of the Big Chestnut", the object being to straighten out the lines of the division, and thus shorten the distance to the picket line. The two companies were left behind to care for the Fort, and I was detailed to remain with them. We did not join the main body for ten days, and when I reached the regiment again I found our horses had arrived, which relieved me of the further necessity of doing duty on foot. (Mem.—My horse arrived October 15 with the other horses, and was taken to camp of the Big Chestnut, whence he was sent to me at Fort Ethan Allen the next day.)

The citizens of Buffalo presented Colonel Bidwell his horse, which was a fine solid fellow just suited to his master's wants. Mine was purchased of Bridges & Millar, of Cowlesville, and was bred by Sargeant. He was a pure black, six years old, and I rode him all through the service, fetching him home with me when I returned. Our horses were all sent to us without cost of transportation, the citizens of Buffalo looking after that; and we presented the man who brought them, or rather superintended their transit, a gold headed cane, as he would accept no compensation in money for his service. I regret that I have forgotten his name.

On October 24 the division was reviewed by General Smith on a large field near his headquarters, which was the first review

I had yet seen. General Stevens was ordered to South Carolina about this time, which left Colonel R. P. Taylor, of the 33d N. Y. V., temporarily in command of the 3d Brigade, by virtue of his seniority of rank. General J. M. Brannan was, however, soon assigned to its command.

On the 26th General Hancock, of the First Brigade, reviewed the command, consisting of fourteen regiments, two batteries, and a troop of cavalry. A sham battle followed the review, and the 49th was complimented by the General on its good appearance and discipline.

About this time the name of the camp was changed in general orders, from Camp Big Chestnut (so named from a tree) to Camp Griffin, in honor of Captain, afterwards General, Griffin whose battery had shelled Lewinsville a few weeks before from a point near where General Smith's headquarters now were, and drove the enemy out. The object of the change was to make the name of the camp uniform throughout the whole division, a division being considered a unit so far as the present organisation of the army of the Potomac was concerned. On October 28 the 21st regiment paid us a visit with its entire field and staff, headed by the Union Cornet Band. We were all very glad to see the "Boys", with many of whom I had a personal acquaintance. The surgeon, Dr. Wilcox, was one of the prominent Buffalo physicians, and I was particularly glad to meet him again, as well as many other officers and men of the command. After remaining with us about an hour and a half they started back to their own camp on Munson's Hill, amidst the huzzas of a thousand friendly voices.

On October 21 we were mustered into the U. S. service by Captain R. B. Ayers, U. S. Army, afterwards Major General Ayers. The muster was, however, to date back to the regular dates of our respective enlistments; and on the 31st we were inspected and mustered for pay—the first time we were put through this ordeal, but which was hereafter to occur at the end of every second month. The inspecting officer must, at this time, call every man's name, check those present, inspect the arms accoutrements, clothing and quarters, the rolls with this certificate attached being the Paymaster's voucher. After the muster Colonel Alberger, Captain Wheeler, and myself went to Falls Church for a ride, and enjoyed the scenery very much. The old church was built of red brick imported from England, the same as Pohick Church at Mount Vernon, and other churches in Virginia.

During these days I frequently visited Washington, which was about ten miles from our camp, sometimes remaining all night with friends in the city, but generally returning the same day with stores, medicines, and camp equipage for the hospital, etc. The requisitions for these articles were made by the Surgeon, approved by the Colonel and the Brigade Commander, when they were ready to be taken by somebody to Washington to be filled. This latter duty fell to me as I have just hinted, and I usually enjoyed going to the city very much indeed; but, while it afforded diversion from its variety, it was often very fatiguing, not to say sometimes perplexing, and I generally returned in the evening tired and hungry, getting a sound sleep, however, as compensation therefor. The regular monthly return of sick was also to be made up at this time, the first we had made, and this duty fell to my lot as junior medical officer. It was a perplexing thing to do at first, but I soon "got the hang" of it, and didn't mind it as much afterwards.

November 1, 1861, saw us fairly established in the full humdrum ways of camp life. I do not mean by this that we were idle,—far from it. But by this time we had gone through those very necessary early experiences of marching, drilling, inspections, and reviews; of requisitions, reports, musters, and picketing; so we were now sufficiently acclimated and habituated to the military service, to make us an effective factor in the autonomy of the division. We had a good hospital in excellent working order, and ours was reputed the healthiest regiment in the division. The men were happy in the confidence of their officers, and realised the fact that they would be well cared for if sick, or otherwise disabled. We had lost one officer, Captain Dickenson, of Chautauqua County, who was seized with an apopletic fit in camp in October. I took him to Georgetown, and placed him in the Seminary Hospital, where he died October 10. He was, I believe, the oldest officer in the regiment, a genial gentleman, and we all felt saddened by his sudden and untimely death.

The Rev. John Bowman, of Niagara County, who had been appointed Chaplain, reported on November 3, and preached to the regiment for the first time the same afternoon, it being Sunday. He was a stranger to most of us, but a man of some ability, and did fairly well while he remained with the regiment. He resigned April 13, 1862, while we were before Yorktown.

We received our first pay on November 8, it being for a month and a half, from September 16 to October 31 inclusive, and it required 38,000 dollars to pay the regiment. My own pay for the same period amounted to $196.24, and it was given to me in gold. Out of the sum I paid all the expenses I had incurred on account of entry into the service, including the cost of horse equipments, etc., retained $25.00 for current expenses, and sent $40.00

to my wife. When everything is considered in regard to the matter of high cost of living, as well as of all articles needed by an officer, this may be regarded as a pretty good reckoning.

All the indications now pointed to the fact that we would probably pass the winter in our present position, and everybody, therefore, began to prepare for a greater degree of comfort. I received another tent that I pitched in the rear of the first one, and was thus afforded two comfortable communicating rooms, each 9x9 feet—the ordinary wall tent size. Our officer's mess was established under one roof, i. e., we built a large log house, roofed in with canvas, and the officers all took their meals together in that building, each paying a pro rata share that was determined by a committee, as in an ordinary club. We were thus enabled to have a greater variety of food at much less expense than if we had broken up into several smaller messes.

(*To be continued.*)

## Figures and Tables

**Figure f1:**